# Association between Overweight, Obesity and the Prevalence of Multimorbidity among the Elderly: Evidence from a Cross-Sectional Analysis in Shandong, China

**DOI:** 10.3390/ijerph17228355

**Published:** 2020-11-12

**Authors:** Fangfang Hu, Lingzhong Xu, Jinling Zhou, Jiao Zhang, Zhaorong Gao, Zhuang Hong

**Affiliations:** 1 NHC Key Laboratory of Health Economics and Policy Research, Cheeloo College of Medicine, Shandong University, Jinan 250012, China; hufang1218@yeah.net (F.H.); zhoujl@sdu.edu.cn (J.Z.); zhangjiao5263@163.com (J.Z.); gaozr20160104@163.com (Z.G.); hongzhuang@mail.sdu.edu.cn (Z.H.); 2 School of Public Health, Cheeloo College of Medicine, Shandong University, Jinan 250012, China; 3 Center for Health Economics Experiment and Public Policy Research, Cheeloo College of Medicine, Shandong University, Jinan 250012, China

**Keywords:** elderly, males and females, multimorbidity, obesity, overweight

## Abstract

(1) Background: Multimorbidity, defined as the occurrence of two or more chronic diseases, is a global public health problem which has a significant negative impact on individuals, families and the society. The aim of this study was to evaluate the association between overweight, obesity and the prevalence of multimorbidity among male and female older adults; (2) Methods: Cross-sectional data of the 7070 participants from China, aged 60 years and above included in 2017 the Shandong Elderly Family Health Service Survey were analyzed. Multivariate logistic regression analysis was used to examine the relationship between overweight, obesity and the prevalence of multimorbidity in males and females; (3) Results: Among the 7070 participants, of which 40.25% were males and 59.75% were females, the average age of all participants was (69.81 ± 6.45) years old. The prevalence of multimorbidity in older adults was 34.71%, and the overweight and obesity rates were 39.25% and 16.89%. Among the male elderly, the likelihood of multimorbidity was more than two times higher among the obese than the normal BMI population (OR: 2.14; 95%CI: 1.63–2.82). A less strong association was found in the overweight male older population (OR: 1.43; 95%CI: 1.18–1.74). In the females, compared with individuals with normal BMI, the risks for incident multimorbidity were high in the overweight and obese groups, with odds ratios of 1.42 (95%CI: 1.21–1.65) and 1.81 (95%CI: 1.51–2.17), respectively. (4) Conclusions: In this study, overweight and obesity had the strongest association with the prevalence of multimorbidity among Chinese older adults, and the associations were different between the male and female elderly. The prevalence of multimorbidity might be effectively prevented by controlling body mass index. Encouraging the elderly to eat the recommended amount of vegetables and fruits, walk at least 30 min a day and have enough sleep to maintain a healthy weight.

## 1. Introduction

China is facing severe issues and challenges because of its rapid process of population aging and continued increase in the size of the older population over a long period of time [1]. According to the China Statistics Yearbook, by the end of 2019, the population over 60 years old in China will have reached 254 million, accounting for 18.1% of the total population. An Analysis Report of National Health Service Survey in China, 2013, showed that the prevalence rate of chronic diseases was 24.5%, and most of the elderly deaths were caused by chronic diseases. Chronic diseases have become prominent social problems and major public health problems among Chinese older adults. In particular, the prevalence of multimorbidity has become a major challenge for chronic disease control [2]. The definition of multimorbidity used by researchers and clinicians was developed by the World Health Organization: two or more chronic diseases coexist, each of which must be a non-communicable disease (NCD), mental health disorder or long-term infectious disease [3].

Multimorbidity was more prevalent in older adults (aged 60 or more) and was a common phenomenon in this age group [4]. The previous study reported that 28% of Americans have multimorbidity of chronic diseases, accounting for two-thirds of healthcare spending [5]. According to a Serbian study, the overall prevalence of multimorbidity was higher than the overall prevalence of a single disease (26.9% vs. 20.7%) among aged ≥20 years [6]. The results of a Chinese study showed that more than half of Chinese people aged 70 or older had multimorbidity [7]. A national study found that 28.58% of the older people suffered from multimorbidity [8]. It can be seen that the prevalence of multimorbidity in the elderly is severe, which has become a major public health problem in China [9,10].

Obesity is recognized as a serious problem and is conventionally categorized on the basis of body mass index (BMI). The increase in BMI is a risk factor of multimorbidity. In many people, the average body mass index has risen by a few percentage points, which has exacerbated concerns about the health effects of increased obesity. Studies at home and abroad have shown that obese people have a high risk of death due to liver cirrhosis, stroke, cardiovascular disease, cancer and trauma [11,12]. The study has shown that the prevalence of multimorbidity in overweight and obese people over 60 years old is 2.5–3.0 times that of the general population [13]. Previous studies have suggested that the risks of stroke, cardiovascular and cerebrovascular diseases, diabetes and malignant tumor were higher than those of normal weight older people [14]. Available research has indicated that prevention of overweight and obesity in the elderly is the key to control the burden of chronic diseases [15]. In exploring the association between BMI and multimorbidity, previous studies reported different findings for males and females. For example, a study in older adults showed significant differences between genders in the estimates of NCDs and in the association between NCDs, multimorbidity and BMI [16]. However, another study in Serbian adults did not support the gender differences [6]. In conclusion, previous studies have found that overweight and obesity are positively associated with multimorbidity in the elderly, but there are different views on the differences between different gender groups. Thus, we aimed to explore the association between overweight, obesity and the prevalence of multimorbidity among male and female older adults in this study.

## 2. Methods

### 2.1. Study Participants and Data Collection

This was a cross-sectional study based on data from 2017 the Shandong Elderly Family Health Service Survey. The detailed sampling and quality controlling regulations have been published elsewhere [17]. This survey was carried out in Weihai, Weifang and Heze in August 2017. These three cities belong to the eastern, central and western parts of Shandong Province, which represent the social and economic development level of different regions. The respondents were people over 60 years old, who were interviewed face to face in their homes. All the measures were self-reported. The main purpose of this investigation was to collect data concerning the basic situation of families and individuals, lifestyle, disease status and medical services through a multi-stage random sampling strategy, questionnaire and structured interview method. In total, the survey included 3 cities in Shandong Province, covering 6 counties (districts), 18 towns (streets), 108 villages (residences), and 7088 older respondents. Of these, 18 were unable to complete an individual questionnaire. In this study, we analyzed data from the final sample of 7070 older people. The protocol for this study was approved by the Ethical Committee (No. 20170110) of the School of Public Health, Shandong University. The investigation was conducted after the acquisition of informed consent of all participants.

### 2.2. Dependent Variable

Multimorbidity, which is measured as the number of chronic diseases, was used to estimate the physical condition of the individuals. We defined multimorbidity as two or more than two chronic diseases occurring in the same individual. In the survey, the interviewees were asked whether they had a chronic disease which had been diagnosed. If they answered “yes”, we continued asking them which chronic disease they had. Then we would obtain the true self-reported data about a number of chronic diseases. These diseases included in the survey: hypertension, diabetes, coronary heart disease, chronic obstructive pulmonary disease, cancer, asthma and others. We classified dependent variables into two categories in this study: individuals with less than two chronic diseases and individuals with two or more chronic diseases.

### 2.3. Independent Variable

We used body mass index (BMI) to serve as an indicator of the degree of obesity. According to the principle of informed consent, an informed consent form was signed with the subjects. The physical investigation was conducted in strict accordance with the “anthropometry method” [18]. The investigators measured the height and weight of each respondent. BMI was calculated by dividing body weight in kilograms by the square of height in meters and categorized into four categories. Values between 18.5 and 23.9 kg/m^2^ were considered to be normal; individuals with BMI between 24 and 27.9 kg/m^2^ were considered to be overweight, while those with BMI ≥28 kg/m^2^ were considered to be obese. Those with a BMI <18.5 kg/m^2^ were considered to be underweight [19].

### 2.4. Covariates

The covariates of this study included demographic variables (gender, age, residence, educational level, self-rated economic status), lifestyle-related variables (smoking status, alcohol consumption and exercise situation (per day)), physiological variables (self-rated health). All variables were categorical. According to age, it could be categorized into young aged people (60–69 years old), middle-aged people (70–79 years old) and old people (≥80 years old). Residence was categorized into rural and urban areas. According to the number of years of self-reported education, educational level was categorized into illiteracy, primary school, junior high school, high school or above. Self-rated economic status was divided into four groups: wealthy and not worried about livelihood, not wealthy but not worried about livelihood, not wealthy and worried about livelihood, poor and worried about livelihood. Responses to both smoking status, alcohol consumption were categorized into “never”, “former” or “current”. According to exercise time a day, exercise situation was categorized into never, less than 20 min, more than 20 min less than 40 min and more than 40. Finally, self-rated health was divided into three grades: good, normal and bad.

### 2.5. Statistical Analyses

We first divided the individuals into two gender groups: males and females. Then we conducted a descriptive statistical analysis of all variables. The results were presented as percentages. Continuous and categorical variables were tested with the χ^2^ test and *t*-test when appropriate. Chi-square tests were also used to compare the distribution of multimorbidity in different BMI groups among male and female elderly adults. On the basis of the above descriptive statistical analysis, multivariate logistic regression analysis was used to estimate the associations between overweight, obesity and multimorbidity, revealing ORs and 95%CIs. The regression models were adjusted for age, residence, education level, self-rated economic status, smoking status, alcohol consumption, exercise situation (per day) and self-health status. SPSS V.25.0 (IBM) was employed to run all statistical analysis. We considered the results to be statistically significant when *p*-value was less than 0.05.

## 3. Results

### 3.1. Descriptive Statistics

Table 1 showed the socio-demographic characteristics of the study participants. The study sample consists of 7070 individuals, of which 40.25% were males and 59.75% were females. The average age of all participants was (69.81 ± 6.45) years old. In contrast to the elderly males, females—the majority of women were 60–69 years old—had an educational level below primary school. The proportion of self-rated health for men was higher than that of women. In terms of chronic diseases, the proportion of older women (37.83%) was higher than that of men (30.08%). Moreover, the rate of overweight in women was higher than that in men, and so was obesity (χ^2^ = 160.568, *p* < 0.001). In addition to residence and exercise situation (per day), the prevalence of multimorbidity was significantly different according to age, gender, education level, self-rated economic status, alcohol consumption, smoking status, self-rated health.

### 3.2. Prevalence of Multimorbidity by Body Mass Index (BMI) Categories

Table 2 shows the prevalence of multimorbidity by body mass index (BMI) categories. The prevalence of multimorbidity was 36.61% and 44.64% among the overweight obese older population. For the obese older women, nearly 46% demonstrated multimorbidity, and about 40% of men were classed as having multimorbidity. The increase in BMI reflected the increase in the prevalence of multimorbidity, whether older men or older women. However, it was more obvious in the female elderly. Among obese people, the prevalence of multimorbidity was significantly higher than that of overweight people, both in the male (40.62% vs. 32.45%) and female (46.11% vs. 39.18%) elderly groups.

### 3.3. Association between BMI Categories and the Prevalence of Multimorbidity in Male and Female Older Adults

Table 3 shows the results of logistic regression analysis for different genders, which estimated the association between BMI status and the prevalence of multimorbidity. Statistically significant associations were observed between overweight and obesity and multimorbidity, which were statistically significant at the 1% level. With the increase in BMI, the risk of multimorbidity also increased. Compared with the elderly with normal BMI, overweight and obese older people were more likely to have multimorbidity. The highest OR values were found in the obese elderly, both in males and females. Among the male elderly, after multivariable adjustment, the likelihood of multimorbidity was more than two times higher among the obese than the normal BMI population (OR: 2.14; 95%CI: 1.63–2.82). Being overweight (OR: 1.43; 95%CI: 1.18–1.74) was associated with significantly higher likelihood of having multimorbidity as compared to being in the normal weight category. With regard to the female elderly, the risk of multimorbidity in those who were obese was 1.81 times higher than those with a normal BMI (OR: 1.81; 95%CI: 1.51–2.17). Overweight (OR: 1.42; 95%CI: 1.21–1.65) had a similar risk for multimorbidity as males. The influence of obesity on the incidence of multiple chronic diseases was greater than that of overweight. The potential gender differences in obesity measurement and incidence rate were analyzed, but there was no difference between the two results.

## 4. Discussion

The goal of this study was to estimate the association between overweight, obesity and multimorbidity among the adults. As far as we know, this was one of the first studies on the relationship between overweight, obesity and the prevalence of multimorbidity through different gender groups. We controlled for confounders, which included demographic variables (gender, age, residence, educational level, self-rated economic status), lifestyle-related variables (smoking status, alcohol consumption and exercise situation (per day)), physiological variables (self-rated health).

We found that the prevalence of multimorbidity was quite high (34.71%) among the Chinese older adults, and the prevalence of multimorbidity was higher in females than in males in our sample. In addition, prevalence of multimorbidity was 1.29 times higher among obese people than the overall population (44.64% vs. 34.71%). A previous study showed that older people with multimorbidity were more common than those with only one chronic disease [20]. Our results were consistent with them. Many studies on the prevalence of multimorbidity have been conducted [21,22,23]. These studies come from different countries, including different data sources, data collection methods and the number of chronic diseases included in the analysis. As for the collection of data, some studies come from the general population [24], while others come from medical institutions databases [25]. Included in these, is a special study on multimorbidity in women [26]. Although the prevalence of multimorbidity in our study was different from other studies, it was considered common to suffer from multimorbidity. However, the previous study in China found that multimorbidity was present in 43.6% of respondents from the sample population [27]. The prevalence of multimorbidity was higher than that of our research, probably because the survey data were of national origin. In addition, a study in a developing country also showed that the prevalence rate of multimorbidity in women is higher than that in men for people over 60 years old [28].

The analysis of 7070 adults over the age of 60 showed that overweight and obese older adults had a higher risk of multimorbidity than normal weight. However, after accounting for some variables, the associations of overweight and obesity with multimorbidity remained strong. Overweight and obese older people were generally more likely to suffer from multimorbidity than normal weight older people. This result was similar to the finding of a national study—overweight and obesity were the risk factors of multimorbidity [29]. Some findings confirm that obesity prevalence is positively correlated with the prevalence of multimorbidity, and obesity was more common in patients with chronic diseases in the sample population we considered [30,31]. We also found that overweight and obesity were independent risk factors for multimorbidity. Our study may have important significance in delaying the possibility of multimorbidity through early and more effective intervention of overweight and obesity. In our study, whether male or female, all ORs for the prevalence of multimorbidity in all BMI categories were higher than other economically-advanced countries, such as Canada and Brazil [26,32]. When we compared these with low and middle-income countries, ORs in all BMI categories were lower than them [33]. We suspected that these countries were limited in the study of multimorbidity. In contrast, the studies in developed countries focused on detailed investigation of other determinants of multimorbidity, including obesity. The differences observed between this study and those studies requires further consideration of other potential risk factors which could lead to multimorbidity.

A previous study found that there were significant gender differences observed in the occurrences of these multimorbidity combinations, and the numbers of women with triad combinations of chronic diseases were generally higher than for men with the same morbidity triads [27]. Although the prevalence of multimorbidity in this study was different from other studies, it was consistent with the other findings. It indicated that the prevalence of multimorbidity was common in older people, and should attract more attention, especially in the female elderly group. Beyond this, we also found that there were some different patterns between male and female elderly people. More specifically, overweight and obese older males were more likely to suffer from multimorbidity than overweight and obese older female adults. After controlling the confounding factors, this phenomenon still existed. Our results supported the view that overweight and obesity are associated with increased risk of multiple diseases, not with potential confounders [34]. Although we found that obese women have a higher proportion of chronic diseases than obese men, obese men have a higher risk of chronic diseases than women, which may be related to the higher life expectancy of women than men. In the current study, the exact cause of gender differences in BMI and chronic diseases is not clear. However, it is well known that obese men are at a higher risk of developing chronic diseases, and were are more likely to develop chronic diseases than obese women [35,36]. One possible reason for the differences between BMI and incidence rate is that male and female elderly people have different body functions. With the onset of age, the body composition of different genders will also change, and there will be differences, which are worthy of further discussion. Based on these findings, we recommend using intelligent electronic devices to record height and weight.

Our study benefited from a number of strengths. In terms of practice, the research results have practical significance and application value. Additionally, the use of reliable data from the 2017 Survey of the Shandong Elderly Family Health Service, makes our research more convincing. At present, there are no such large sample studies on the relationship between overweight, obesity and the prevalence of multimorbidity in male and female Chinese elderly. However, there were also many limitations considered in our study. Firstly, the subjects of this study were elderly adults, over 60 years old, in Shandong Province. We could not represent the elderly in the whole of China. Secondly, the prevalence of chronic diseases in our statistics was self-reported by patients, which might cause recall bias. Thirdly, although we controlled the important confounding factors of body index, some unobserved factors might still affect our main independent and dependent variables. Lastly, data for this study were derived from a cross-sectional design survey. Our research design did not allow for a causal relationship between obesity and disease. However, it was unreasonable to construct a reverse relationship in which a disease was the cause of obesity.

## 5. Conclusions

In conclusion, the prevalence of multimorbidity among Chinese aged ≥60 years was high and varied by gender. The associations between overweight, obesity and multimorbidity were different between the male and female older adults. It has been previously shown that overweight and obesity are associated with the development of many chronic diseases, but our findings further clarify the association between overweight, obesity and the prevalence of multimorbidity by considering different gender groups. The prevalence of multimorbidity might be effectively prevented by controlling body mass index. We should pay close attention to the physical condition of older adults, guide them to develop a healthy and reasonable lifestyle and habits, and encourage them to eat the recommended amount of vegetables and fruits, walk at least 30 min a day and obtain enough sleep to maintain a healthy weight.

## Figures and Tables

**Table 1:** Descriptive characteristics of study participants by gender groups.

Characteristics	Total n (%)	Males n (%)	Females n (%)	*p*
Observations	7070 (100)	2846 (40.25)	4224 (59.75)	
Age (mean; SD)	69.81 (6.45)	70.29 (6.40)	69.48 (6.46)	0.000 ^a^
Age Group				0.000 ^b^
60–69	3706 (52.42)	1386 (48.70)	2320 (54.92)	
70–79	2755 (38.97)	1199 (42.13)	1556 (36.84)	
≥80	609 (8.61)	261 (9.17)	348 (8.24)	
Residence				0.000 ^b^
Rural	5514 (77.99)	2366 (83.13)	3148 (74.53)	
Urban	1556 (22.01)	480 (16.87)	1076 (25.47)	
Education level				0.000 ^b^
Illiteracy	2270 (32.11)	491 (17.25)	1779 (42.12)	
Primary school	2924 (41.36)	1294 (45.47)	1630 (38.59)	
Junior high school	1315 (18.60)	718 (25.23)	597 (14.13)	
High school or above	561 (7.93)	343 (12.05)	218 (5.16)	
Self-rated economic status				0.036 ^b^
Wealthy and not worried about livelihood	1618 (22.89)	696 (24.46)	922 (21.83)	
Not wealthy but not worried about livelihood	4920 (69.59)	1932 (67.88)	2988 (70.74)	
Not wealthy and worried about livelihood	478 (6.76)	192 (6.75)	286 (6.77)	
Poor and worried about livelihood	54 (0.76)	26 (0.91)	28 (0.66)	
Alcohol consumption				
Never	5365 (75.88)	1229 (43.18)	4136 (97.92)	0.000 ^b^
Former	552 (7.81)	527 (18.52)	25 (0.59)	
Current	1153 (16.31)	1090 (38.30)	63 (1.49)	
Smoking status				
Never	5029 (71.13)	886 (31.13)	4143 (98.08)	0.000 ^b^
Former	903 (12.77)	875 (30.74)	28 (0.66)	
Current	1138 (16.10)	1085 (38.12)	53 (1.25)	
Exercise situation (per day)				0.150 ^b^
Never	2719 (38.46)	1137 (39.95)	1582 (37.45)	
Less than 20 min	507 (7.17)	193 (6.78)	314 (7.43)	
More than 20 less than 40 min	1680 (23.76)	673 (23.65)	1007 (23.84)	
More than 40 min	2164 (30.61)	843 (29.62)	1321 (31.27)	
Self-rated health				0.000 ^b^
Good	3782 (53.49)	1610 (56.47)	2172 (51.42)	
Normal	1992 (28.18)	747 (26.25)	759 (29.47)	
Bad	1296 (18.33)	489 (17.18)	390 (19.11)	
BMI (mean; SD)	24.68 (3.69)	23.99 (3.40)	25.15 (3.80)	0.000 ^a^
BMI categories				0.000 ^b^
Underweight	235 (3.32)	122 (4.29)	113 (2.68)	
Normal	2866 (40.54)	1344 (47.22)	1522 (36.03)	
Overweight	2775 (39.25)	1060 (37.25)	1715 (40.60)	
Obesity	1194 (16.89)	320 (11.24)	874 (20.69)	
Number of chronic diseases				0.000 ^b^
0	2208 (31.23)	1024 (35.98)	1184 (28.03)	
1	2408 (34.06)	966 (33.94)	1442 (34.14)	
≥2	2454 (34.71)	856 (30.08)	1598 (37.83)	

^a^*t*-test; ^b^ chi-squared test; SE = Standard Error.

**Table 2:** Prevalence of multimorbidity (%) by BMI categories.

Characteristics		BMI Categories				*p* *
		Underweight	Normal	Overweight	Obesity	
Total	Multimorbidity					0.000
	No	171 (72.77)	2025 (70.66)	1759 (63.39)	661 (55.36)	
	Yes	64 (27.23)	841 (29.34)	1016 (36.61)	533 (44.64)	
Males	Multimorbidity					0.000
	No	87 (71.331)	997 (74.18)	716 (67.55)	190 (59.38)	
	Yes	35 (28.69)	347 (25.82)	344 (32.45)	130 (40.62)	
Females	Multimorbidity					0.000
	No	1028 (67.54)	84 (74.34)	1043 (60.82)	471 (53.89)	
	Yes	494 (32.46)	29 (25.66)	672 (39.18)	403 (46.11)	

* χ^2^; BMI, body mass index.

**Table 3:** Multivariate logistic regression of BMI categories associated with the prevalence of multimorbidity in male and female older adults.

Variables	Males (n = 2846)				Females (n = 4224)			
	Unadjusted		Covariates *		Unadjusted		Covariates *	
	OR (95%CI)	*p*	OR (95%CI)	*p*	OR (95%CI)	*p*	OR (95%CI)	*p*
BMI categories (ref: normal)								
Underweight	1.16 (0.77–1.74)	0.490	1.00 (0.64–1.56)	0.995	0.72 (0.46–1.11)	0.137	0.63 (0.40–0.99)	0.048
Overweight	1.38 (1.16–1.65)	0.000	1.43 (1.18–1.74)	0.000	1.34 (1.16–1.55)	0.000	1.42 (1.21–1.65)	0.000
Obesity	1.97 (1.52–2.54)	0.000	2.14 (1.63–2.82)	0.000	1.78 (1.50–2.11)	0.000	1.81 (1.51–2.17)	0.000

BMI, body mass index; * BMI categories adjusted for age, residence, education level, self-rated economic status, alcohol consumption, smoking status, exercise situation (per day), self-rated health.
